# The Medical Applications of Novel PET Radiopharmaceuticals

**DOI:** 10.3390/ph18101512

**Published:** 2025-10-09

**Authors:** Jonathan Vigne, Florent L. Besson

**Affiliations:** 1 Department of Nuclear Medicine, CHU de Caen Normandie, Normandie Université, UNICAEN, 14000 Caen, France; 2 Department of Pharmacy, CHU de Caen Normandie, Normandie Université, UNICAEN, 14000 Caen, France; 3 INSERM U1237, PhIND, Institut Blood and Brain @ Caen-Normandie, Centre Cyceron, Normandie Université, UNICAEN, 14000 Caen, France; 4 BioMaps, CEA/Inserm/CNRS/Université Paris-Saclay, 91400 Orsay, France; florent.besson@aphp.fr; 5 Department of Nuclear Medicine-Molecular Imaging, Hôpitaux Universitaires Paris-Saclay AP-HP, DMU SMART IMAGING, Hôpital Bicêtre, 94270 Le Kremlin-Bicêtre, France; 6 School of Medicine, Université Paris-Saclay, 94270 Le Kremlin-Bicêtre, France

The landscape of medical imaging is undergoing a profound transformation, driven by the development of novel positron emission tomography (PET) radiopharmaceuticals. These next-generation tracers represent a major advancement in precision diagnostics, offering unprecedented insights into disease processes at the molecular level. PET imaging has long been a cornerstone in the diagnosis and management of cancer, cardiovascular diseases, and neurological disorders. While established tracers such as [^18^F]FDG remain integral to clinical practice, the advent of new radiopharmaceuticals targeting specific cellular and molecular pathways is expanding PET’s diagnostic power. This evolution significantly supports the paradigm of personalized medicine, enabling earlier detection, more accurate diagnosis, and real-time monitoring of therapeutic responses [1,2,3].

Within the field of oncology, prostate-specific membrane antigen (PSMA)-targeted PET agents have revolutionized the management of prostate cancer, and the development of therapeutic analogues has established PSMA as a paradigm of theranostics [4]. Similarly, fibroblast activation protein inhibitors (FAPIs) have rapidly emerged as promising radiotracers for both imaging and therapy. Preclinical and early clinical studies demonstrated high tumor uptake and favorable biodistribution across a wide spectrum of malignancies [5,6]. In neuro-oncology, amino acid tracers such as [^18^F]FET have become essential for glioma diagnosis, grading, and treatment monitoring, outperforming [^18^F]FDG in many settings [7]. Beyond cancer, PET imaging of infection, inflammation, and fibrotic disorders is gaining momentum, with novel tracers enabling earlier and more precise evaluation of disease activity [8,9].

This Special Issue brings together key advances in these areas, with contributions that explore the wide-ranging clinical applications of these novel PET agents, with a particular focus on fibroblast activation protein inhibitors (FAPIs) and amino acid-based tracers such as [^18^F]FET. Rizzo et al. present a meta-analysis on [^68^Ga]Ga-FAPI PET in head and neck cancers, demonstrating its high detection rate for primary tumors and solid performance in lymph node staging [10]. Serumula et al. offer a comprehensive review of FAPI-based theranostics, highlighting its emerging dual role in both diagnosis and therapy, particularly for tumors characterized by low glycolytic activity [11]. Bertaux et al. investigate the prognostic value of [^18^F]FDG-PET/CT in patients with metastatic bladder cancer undergoing immunotherapy, comparing various response assessment criteria, including PERCIST5, imPERCIST5, and PERCIMT [12]. In the realm of neuro-oncological disorders, Robert et al. deliver a comprehensive review of [^18^F]FET PET in the management of gliomas. Drawing from more than 80 studies, their work hightlights the tracer’s clinical value in diagnosis, tumor grading, therapy planning, and post-treatment follow-up [13].

Beyond oncology, Albano et al. provide a systematic review of FAPI PET/CT in non-oncologic conditions, revealing strong diagnostic potential in diseases such as interstitial lung disease, IgG4-related disease, and Crohn’s disease [14].

The contributions in this Special Issue underscore the transformative potential of PET radiopharmaceuticals in advancing personalized and molecular medicine. Whether in oncology, inflammation, fibrosis, or neuroimaging, these agents enhance the precision and scope of PET imaging.
